# Sonographic Evaluations of the Pubic Symphysis at Different Stages of Pregnancy

**DOI:** 10.3390/jcm14113898

**Published:** 2025-06-01

**Authors:** Slawomir Wozniak, Aleksandra Piatek, Bozena Kurc-Darak, Zygmunt Domagala, Friedrich Paulsen, Jerzy Florjanski

**Affiliations:** 1 Division of Anatomy, Department of Human Morphology and Embryology, Wroclaw Medical University, Chalubinskiego 6a, 50-368 Wroclaw, Poland; 2 2nd Department and Clinic of Gynaecology and Obstetrics, Wroclaw Medical University, Borowska 213, 50-556 Wroclaw, Poland; 3 Institute of Functional and Clinical Anatomy, Friedrich Alexander University Erlangen-Nürnberg (FAU), Universitätsstr. 19, 91054 Erlangen, Germany; friedrich.paulsen@fau.de

**Keywords:** pubic symphysis, sonographic examination, sonographic anatomy of pubic symphysis, pregnancy

## Abstract

**Background/Objectives**: The pubic symphysis is formed by the fusion of the right and left pubic bones. The metrics, such as breadth, length, and depth, increase during pregnancy and can be measured and analyzed using standard sonography. Obstetricians require clear and consistent criteria for standard sonography evaluation. **Methods**: Sonographic examinations were performed on a cohort of 225 pregnant women, aged between 23 and 41 years, as part of a prospective observational study. The parameters measured included pubic symphysis entry middle width, intertubercular distance, pubic symphysis width, and pubic symphysis depth. **Results**: The width of the pubic symphysis exhibited the greatest consistency, measuring between 2.2 and 11.3 mm, whereas the depth displayed the highest variability, ranging from 5.4 to 22.6 mm. The measurements most correlated with fetal weight included pubic symphysis entry width (6.5 ± 3.4 mm; *p* ˂ 0.001), pubic symphysis width (6.4 ± 2.9 mm; *p* ˂ 0.001), and depth (14.8 ± 4.8 mm; *p* = 0.03). The intertubercular distance exhibited the strongest correlation with maternal age (15.1 ± 5.4 mm; *p* = 0.03). In contrast, pubic symphysis entry width (6.4 ± 3.3 mm; *p* = 0.02; 6.4 ± 3.4 mm; *p* ˂ 0.001) and pubic symphysis width (6.3 ± 2.6 mm; *p* = 0.01; 6.3 ± 2.6; *p* ˂ 0.001) demonstrated stronger associations with maternal weight and weight gained during pregnancy, respectively. In the singular pregnancy group, the width of the pubic symphysis exhibited significant correlations with fetal weight categories: under or equal to 1000 g (4.56 ± 1.5 mm; *p* = 0.02), 1001–2000 g (5.51 ± 2.6 mm; *p* = 0.02), and more than 3000 g (7.3 ± 3.9 mm; *p* = 0.02). Pubic symphysis entry width is significantly correlated with fetal weight in the range of 1001–2000 g (5.5 ± 3 mm; *p* = 0.02) and fetal weight exceeding 3000 g (7.4 ± 3.9 mm; *p* = 0.02). In singular pregnancies, statistically significant differences were noted in intertubercular distance (15.9 ± 7.2 mm vs. 13.4 ± 6.2 mm; *p* = 0.03) when comparing fetuses weighing 2000 g or less between nulliparous and multiparous women. **Conclusions**: Fetal and maternal weight were the primary parameters that were positively correlated with these measurements. The term ‘pubic symphysis entry’ is proposed to describe a trapezoidal space situated superior to the pubic symphysis disc, delineated by an imaginary line connecting the bilateral pubic tubercles.

## 1. Introduction

The pubic symphysis (PS, Latin *symphysis pubica*) is a vital anatomical structure that plays an important role, especially during pregnancy and childbirth. It consists of a fibrocartilaginous disk made of hyaline and fibrous cartilage that connects the right and left bony symphysial surfaces, reinforced by the superior and inferior pubic ligaments [1,2,3,4,5]. The pubic tubercles (PTs) are significant anatomical landmarks located approximately 2 cm from the median plane. The characteristics of the PS vary with age and differ between genders [6]. The anatomy of the PS is clinically significant during sporting activities and pregnancy, particularly during labor and delivery [2,3,7]. Unrecognized severe pathological PS disorders may result in anterior pelvic instability and/or inguinal discomfort [5].

The clinical manifestations of these conditions include pelvic pain and/or ambulation difficulties. One of the most significant consequences is PS diastasis. These matters necessitate consideration, as the documented incidence of non-traumatic diastasis fluctuates significantly, ranging from 1 in 330 to 1 in 30,000 pregnancies. Diagnosis is determined by a PS widening of more than 10 to 13 mm as observed in radiological imaging [8,9].

During pregnancy, the PS undergoes significant changes due to hormonal influences, especially from relaxin, which enhances its width and flexibility to aid in birth [10]. The width of the PS steadily increases from approximately 4 mm at 8 weeks to 7 mm at term [2,8]. However, the correlation between PS morphology and pregnancy-related symphyseal pain is ambiguous, impacting 3–8% of pregnant women [1,11,12,13,14].

Ultrasonography (US) is the preferred method for evaluating the PS due to its ease and effectiveness [15,16,17,18]. Severe complications, such as PS rupture, though rare, can be life-threatening and may require surgical intervention [19,20,21].

The convergence of results obtained from US and other radiologic techniques, including magnetic resonance imaging (MRI), in symptomatic and asymptomatic postpartum patients fails to consistently distinguish between normal and pathological findings. Nonetheless, US or MRI can differentiate among symphyseal contusion, diastasis, and rupture [6,22,23,24]. This clinical issue is of considerable significance and is examined by obstetricians but also by anatomists, orthopedists, and sports physicians alike [4,23,25].

This study seeks to assess how the morphology of the PS, as observed through sonography, correlates with fetal weight and the basic anthropometric measurements of pregnant women. These insights have the potential to improve clinical practices and outcomes.

We introduced the term ‘Ps entry’ to refer to a trapezoid-shaped space located superior to the PS disc. The longer horizontal distance is situated superiorly between both the right and left PTs, while the shorter one is situated inferiorly along the superior border of the PS disc. The obliquity on both sides runs from the right and left PTs to the right and left lateral-superior angles of the pubic PS disc, respectively.

## 2. Materials and Methods

Ethical issues

The provisions of the 1964 WMA Declaration of Helsinki (including subsequent revisions) and Good Clinical Practice served as the foundation for conducting our observational study. All participants provided written consent to participate in this study. The project design was approved by the local ethics committee of the Medical University of Wroclaw (IRB Decision No. 16/2021).

Sonographic examinations, patients

This prospective observational study was conducted from July 2021 to July 2022. Ultrasound examinations were performed using a General Electric E8 ultrasound machine (2D/3D/4D Imaging; HDlive advanced 4D ultrasound imaging; M-Mode; M-Color Flow; General Electric, Boston, MA, USA) on a cohort of 225 pregnant women aged between 23 and 41 years. Women under 18 years of age, those with a history of major systemic bone diseases, those currently taking steroids, those with pelvic trauma, or those who refused consent were excluded. The inclusion criteria were pregnant women over 18 years of age, at different stages of singular pregnancies, including both nulliparous and multiparous women. During the examination, participants were positioned either supine or in a left-sided decubitus position. A 3.4–5.0 MHz volumetric transducer was used for imaging. A thin layer of gel (Aquasonic 100, Parker Laboratories, Fairfield, CT USA) was applied to the transducer head, which was positioned at a 90° angle to the skin at the pubic hairline and then angled toward the anterior pelvic wall. The frequency, focus, and gain were adjusted to optimize scan quality and enhance visibility.

Each participant underwent a single scan during the study. Gestational age ranged from 21 to 42 weeks, with fetal weights varying from 434 to 4100 g.

The evaluations were meticulously performed by an experienced examiner (JF), who has over 35 years of expertise in ultrasound imaging and conducts approximately 2500 examinations annually. The examiner’s proficiency is certified by the Polish Society of Gynaecologists and Obstetricians and the Fetal Medicine Foundation (London, UK).

Ultrasound Visualization

**Figure 1:**
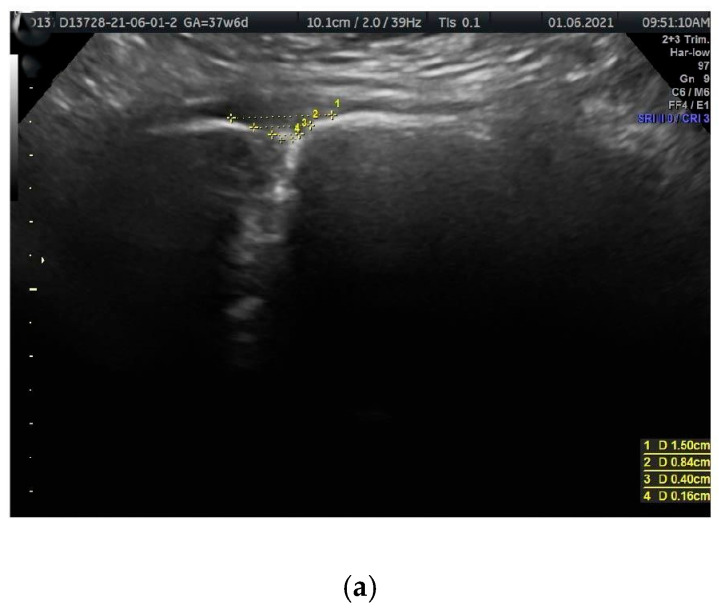

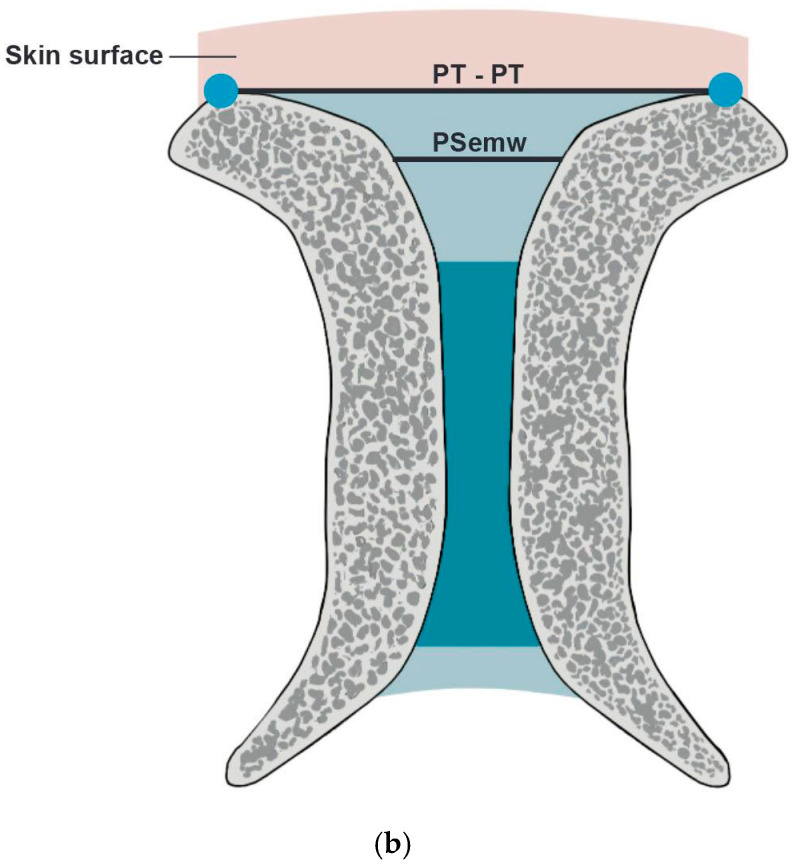
(**a**,**b**) The sonographic transducer was placed on the superior border of the anterior bony wall of the pelvis, parallel to the anterior abdominal wall. (**a**) Measured parameters displayed in the sonographic scan. Distance 1 = PT-PT (intertubercular distance); distance 3 = PSemw (PS entry middle width). (**b**) A corresponding schematic drawing exemplifying the measurements of PS parameters.

**Figure 2:**
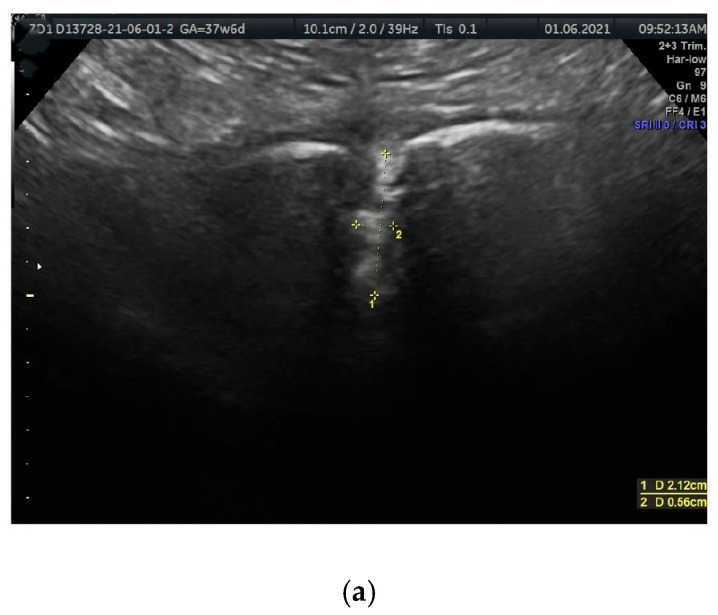

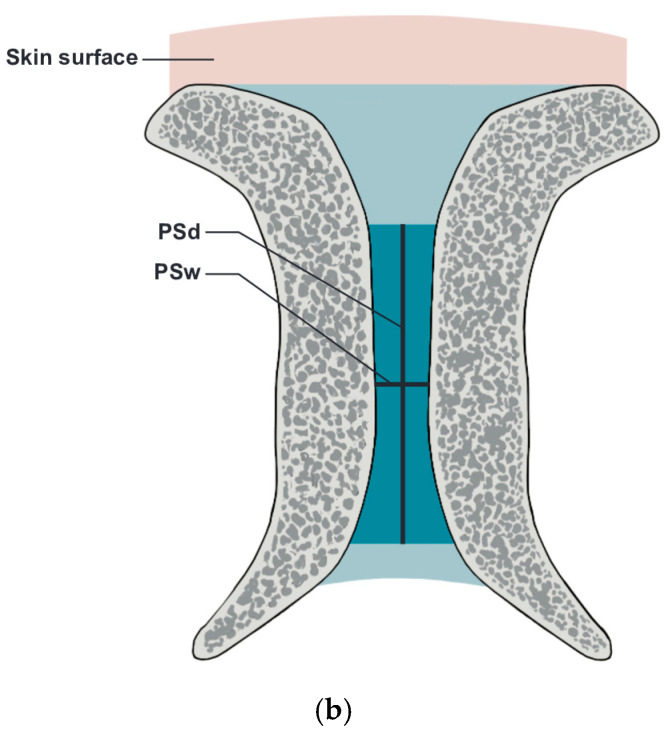
(**a**,**b**). The sonographic transducer was placed on the superior border of the anterior bony wall of the pelvis, parallel to the anterior abdominal wall. (**a**) Measured parameters displayed in the sonographic scan. Distance 1 = PSw (PS width); distance 2 = PSd (PS depth). (**b**) A corresponding schematic drawing exemplifying the measurements of PS parameters.

Ultrasound visualization was performed using coronal (frontal) planes, with the transducer directed from the superior margin to the inferior margin of the anterior pelvic bony wall, parallel to the anterior abdominal wall. In this plane, the PS resembles a funnel that gradually narrows inferiorly (Figure 1a,b and Figure 2a,b). The US scan consists of a wider, quadrangular part, which we propose to name the PS entry, and the proper, rectangular PS disc. The PS entry is bordered superiorly by the intertubercular line, which runs between the right and left PTs. The superior border of the PS disc forms the inferior border of this entry. The lateral borders, both right and left, are defined by lines connecting the PTs to the lateral-superior angles of the disc.

The PS entry exhibits a hyperechoic (bright on ultrasound) appearance, indicating that the tissues in this region—including the superior pubic ligament, connective tissue, and fat—strongly reflect ultrasound waves. Variability in shape among different women may be attributed to individual anatomical differences or changes that occur over time.

Measurements (in mm)

The measured parameters are illustrated in Figure 1a,b and Figure 2a,b.

Horizontal parameters

1. PS entry middle width (PSemw): The distance measured at the midpoint of the PS entrance.
2. Intertubercular distance (PT-PT): The span between the right and left PTs.
3. PS width (PSw): The span between the right and left symphysial surfaces, measured at the midpoint of the PS. Another term for this is the PS width across the PS.

Vertical parameters

1. PS depth (PSd): The distance between the proximal and distal borders of the PS disc.

Statistical analysis

The normality of variables was assessed using the Kolmogorov–Smirnov test (with Lilliefors correction). Justification for the use of non-parametric tests—The data do not meet the assumptions of normal distribution, and the sample size is small. Data for continuous variables are presented as range (min; max) and mean ± standard deviation (SD), while categorical variables are summarized as N (%). The Kruskal–Wallis one-way analysis of variance by ranks/H/was employed for estimations. Analyses were conducted using the Statistica 13 software package (StatSoft Polska, Cracow; Wroclaw Medical University license). The Pearson correlation coefficient test was used to evaluate the correlation between parameters. Statistical significance (SS) was established at *p* = 0.05.

Repeatability and reproducibility

In a preliminary study, we repeated assessments in 20 female patients who underwent routine post-delivery examination. Two experienced sonographers, AP and JF, analyzed the parameters, and JF subsequently re-examined the patients. The differences in measured parameters were less than 1% for every measurement.

This study builds upon the PS sonographic investigation previously presented at the 116th Annual Meeting of the German Anatomical Society [26].

## 3. Results

**Figure 3:**
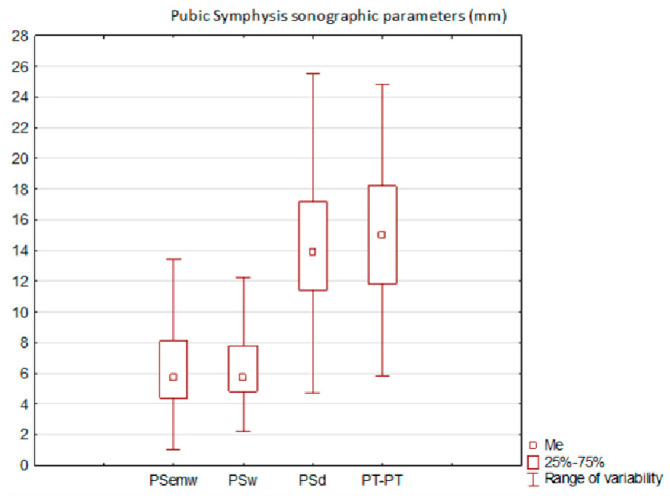
Whole group measured parameters. Me, mean; PSemw, PS entry middle width; PSw, PS width; PSd, PS depth; PT—PT, Intertubercular distance.

The most consistent parameter in the measurements was PSw, ranging from a minimum of 2.2 mm to a maximum of 11.3 mm. In contrast, the most variable parameter was PSd, with values ranging from 5.4 mm to 22.6 mm. The collective results are presented in Figure 3.

Interestingly, the parameters most correlated with fetal weight were PSemw and PSw, with Pearson correlation coefficients of 0.28 and 0.29, respectively. Additionally, PSemw showed the strongest correlation with weight gain in women, with a Pearson correlation coefficient of 0.24.

We present PS measurements in Table 1 (in relation to fetal weight) and Table 2 (in relation to female parameters).

Interpretation of results

Fetal weight vs. PS parameters:

- Generally, heavier fetuses are associated with increased PS entry and PS parameters, except for PT span.

Female age vs. PS parameters:

- Older females tend to have longer PT-PT distances. No statistically significant correlation was found with other PS parameters.

Weight before pregnancy vs. PS parameters:

- Heavier women showed wider PS entry and PS discs, along with PT-PT distance. Similar trends were observed with weight gained during pregnancy.

Non-significant correlations:

No statistically significant correlations were identified for the following:

- Height: Patients’ mean height (166.5 ± 5.7 cm) versus PS parameters (PSemw: 6.6 ± 3.4 mm, PSw: 6.5 ± 2.8 mm, PSd: 14.6 ± 4.8 mm, PT-PT: 15.3 ± 5.4 mm).
- BMI during pregnancy: Patients’ mean BMI (24.2 ± 4.2) versus PS parameters (PSemw: 5.6 ± 2.4 mm, PSw: 6.0 ± 2.4 mm, PSd: 14.3 ± 4.1 mm, PT-PT 15.3 ± 5.4 mm).

Each PS parameter is presented as mean ± sd, measured in mm.

We conducted a more in-depth analysis of PS parameters in relation to

- different fetal weights in singular pregnancies (Table 3)
- nulliparity and multiparity (Table 4).

**Interpretation of results**

- Singular Pregnancies: Fetal Weight:
  PSemw: Statistically significant differences were observed for fetuses weighing 1001–2000 g.
  PSw: statistically significant differences were revealed between
  1. Fetuses weighing under 1000 g versus those weighing more than 3000 g
  2. Fetuses weighing 1001–2000 g versus those weighing more than 3000 g
  No statistically significant differences were found for PSd or PT-PT.

**Interpretation of results**

Singular Pregnancies—Nulliparity vs. Multiparity: Fetal Weight

Statistically significant differences in PT-PT were observed when comparing fetuses weighing 2000 g or less between nulliparous and multiparous women.

## 4. Discussion

The widening (extending, upscaling) of the PS begins at around 8–10 weeks of gestation and progresses steadily. Becker et al. used ultrasound to measure different aspects of the PS in 30 healthy women who had never given birth, including the narrow PSw, wide PSw, superior pubic ligament length, the distance between the superior pubic ligament and the superior surface of the SP disc, and the length between the PT and the pubic angle [1]. Our proposed measurement of PSemw (6.4 ± 3.3 mm) falls between Becker’s wide (10.1 ± 4.9 mm) and narrow (2.6 ± 0.7 mm) PS distance. We reaffirm our position that the comparison of results is hampered by the imprecise methodology of conducted studies [2]. We decided to measure and analyze parameters other than those assessed by Becker et al. because our measurements were easier to incorporate into routine obstetric examinations compared to those proposed by Becker et al.

Our measurements of the PSw (at the midpoint of the PS) were 6.4 ± 2.9 mm, differing from those reported by Becker et al. for nulliparous women (2.6 ± 0.7 mm) and aligning more consistently with those reported by Bauman et al. (23 patients; sonographic examination;) for trauma patients of both genders, 4.4 ± 1.3 mm [1,18]. Other authors measured the PSw as slightly larger, at 8.3 ± 2.3 mm [27]. These differences can be attributed to variations in patient groups, measurement levels, and examination techniques. Our measurements were conducted in pregnant women, whose pelvic anatomical changes must be considered. This issue warrants further exploration in future research.

We found that heavier fetuses generally correlated with increased PS entry and disc parameters, except for the span between the right and left PTs. Our interpretation is that heavier fetuses are in advanced gestation, so the PS is prepared for birth (wider and deeper), while the distance between the PTs remains stable. This pattern demonstrates pregnancy-related changes in the PS structure rather than basic pelvic morphology.

The distance between PTs was larger in older women, which we attribute to the morphology of older females. No other parameters were age-related.

Additionally, heavier women had wider PS entry and PS disc dimensions, but these were not deeper. We found no statistically significant correlation with the distance between PTs. We observed similar trends with weight gain during pregnancy. These results suggest that weight correlates with the broadening of the PS parameters but not pelvic morphology.

Clinical implications: In heavier fetuses, we should expect a widening of the PS at both the entry and disc. The same phenomenon is observed in heavier females. However, in older pregnant women, we should also anticipate an enlargement of the PT-PT. Moreover, in nulliparous females with fetuses weighing ≤2000 g, we should expect a longer PT-PT distance, but no changes in other parameters. The changes observed in both heavier fetuses and women were linked to the widening of the pelvis. Older women are expected to have a wider pelvis, as indicated by PT-PT elongation. We speculate that nulliparous females exhibit a similar pelvic morphology.

We support researchers’ opinion that further studies are needed to understand changes in PS and its surrounding structures [2,3].

The PS is covered by a group of ligaments—superior and inferior pubic ligaments—which ensure the stability of this connection and provide attachment areas for surrounding muscles, such as the rectus abdominis and pyramidalis [7]. A compartment of subcutaneous tissue is also present in this region. These structures are easily visualized with sonography. The ligaments absorb tensile forces, the hyaline cartilage absorbs compressive forces, and the fibrocartilage absorbs shear forces.

The PS region is a complex structure responsible for and contributing to pubic-related groin pain, not only during pregnancy, which may complicate this ailment [3,28]. Postpartum symphyseal disruption, although rare, is a severe complication that can occur after delivery, and treatment is typically conservative with PS widening lower than 25 mm [29]. Surgery may be necessary in more advanced cases. Nevertheless, the prognosis for subsequent pregnancies remains favorable [8].

According to Zhang et al. (11 pregnant women in 31–40 weeks of pregnancy), different delivery positions might positively affect PS parameters. These authors concluded that thigh hyperflexion and the effect of gravity influenced childbirth outcomes. However, due to the small sample size, no definitive conclusions could be drawn [27]. The PS changes during delivery, and upright delivery posture in women without epidural anesthesia might be beneficial [30]. While this is an important issue, it is not specifically related to PS morphology and was not analyzed in our study.

There is a growing interest in applying dynamic ultrasound imaging for the prompt diagnosis of patients [31].

We selected the described parameters (PSemw, PT-PT, PSw, and PDs) for analysis because they are easy to measure via ultrasound during routine obstetric examinations. These measurements can be repeated in any unit worldwide. The PS parameters are useful for evaluating pregnancy development. The use of ultrasound fetal measurements—including fetal weight and standard parameters such as head circumference, abdominal circumference, femur length, and biacromial diameter—is gradually expanding, and there is growing interest in these aspects, which are crucial in routine obstetrics [32,33,34].

Major limitations: We did not analyze pregnant females at subsequent stages of pregnancy or after labor in long-term follow-up. All primary study measurements were conducted by a single operator (JF).

Minor limitations: We could not exclude the influence of previous gestations on the analyzed PS parameters in multiparous pregnant females. Additionally, we did not include a control group.

Future research should be conducted on the same group of pregnant females as their gestation progresses, with fetuses gradually becoming more developed and advanced.

The supporting information can be obtained from the Supplementary.

## 5. Conclusions

From a practical point of view, the PS standard parameters should be as follows: PSemw, 6.5 ± 3.4 mm; PSw, 6.4 ± 2.9 mm; PSd, 14.8 ± 4.8 mm; and PT-PT, 15.8 ± 5.3 mm. An increase suggests the risk of diastasis, while a decrease may indicate an improper pregnancy course. Heavier fetuses were associated with increased PS entry and disc parameters. Older females tend to have longer PT-PT distances. Heavier women and those who gained significant weight during pregnancy had wider PS entry and PS discs. In nulliparity versus multiparity, only differences in PT-PT were statistically significant for fetuses weighing 2000 g or less.

## 6. Strengths

We measured and analyzed PS parameters in relation to selected important factors.

## 7. Patents

Future prospective study

Sonographic measurements of body donors and their comparison to measurements after macroscopic preparation are necessary.

## Figures and Tables

**Table 1:** PS measurements in relation to fetal weight.

Pubic Symphysis Sonographic Parameters	Fetal Weight: 2504.3 ± 1014.4 (Mean ± sd) gram	
	Mean ± sd; mm	P; r
PSemw	**6.5 ± 3.4**	**˂** **0.001; 0.28**
PSw	**6.4 ± 2.9**	**˂0.001; 0,29**
PSd	**14.8 ± 4.8**	**0.03; 0.15**
PT-PT	15.8 ± 5.3	0.05; 0.05

PSemw, PS entry middle width; PSw, PS width; PSd, PS depth; PT-PT, Intertubercular distance. Statistically significant *p*-values are shown in bold. P = probability; r = Pearson correlation coefficient.

**Table 2:** PS measurements in relation to female parameters.

Pubic Symphysis Sonographic Parameters	Age ** 30.3 ± 5.3 (Mean ± sd) Years	Weight * 65.1 ± 15.3 (Mean ± sd) kgs	Weight Gained ** (Mean ± sd) 12 ± 5.7 kgs
PSemw	6.5 ± 3.3	**6.4 ± 3.3** ***p* = 0.02; r = 0.16**	**6.4 ± 3.3** ***p* = ˂ 0.001; r = 0.24**
PSw	6.4 ± 2.9	**6.3 ± 2.6** ***p* = 0.01; r = 0.17**	**6.3 ± 2.6** ***p* = ˂ 0.001; r = 0.19**
PSd	14.6 ± 4.9	14.8 ± 4.9	14.8 ± 4.9
PT-PT	**15.1 ± 5.4** ***p* = 0.03; r = 0.15**	15.7 ± 5.3	15.7 ± 5.3

* before pregnancy; ** during pregnancy. PSemw, PS entry middle width; PSw, PS width; PSd, PS depth; PT-PT, Intertubercular distance. Statistically significant *p*-values are shown in bold. P = probability; r = Pearson correlation coefficient;.

**Table 3:** PS measurements in relation to fetal weight in singular pregnancies.

Measured Parameter	Singular Pregnancy: Fetal Weight. Mean± sd mm (No.: 197)				*p* *
	Under/or 1000 g (19)	1001–2000 g (28)	2001–3000 g (70)	More than 3000 g (80)	
PSemw	4.7 ± 1.7 mm	**5.5 ± 3.0 mm** * *p* = 0.02	6.4 ± 3.1 mm	**7.4 ± 3.9 mm** * *p* = 0.02	H = 11.2
PSw	**4.56 ± 1.5 mm** * *p* = 0.02	**5.51 ± 2.6 mm** * *p* = 0.02	6.4 ± 3.1 mm.	**7.33 ± 3.9 mm** * *p* = 0.02	H = 13.6
PSd	13.9 ± 4.7 mm	13.6 ± 4.7 mm	14.8 ± 4.9 mm	15.6 ± 5.2 mm	H = 8.2 *p* = 0.05
PT-PT	15.2 ± 5.1 mm	16.0 ± 5.2 mm	15.3 ± 5.2 mm	15.8 ± 5.4 mm	H = 1.12 *p* = 0.7

* Kruskal–Wallis one-way analysis of variance by ranks/H/; statistically significant *p*-values are shown in bold.

**Table 4:** PS measurements in relation to fetal weight in singular pregnancies of nulliparity vs. multiparity.

Measured Parameter	Singular Pregnancy Mean ± sd mm (No.: 197)						*p* *
	Fetal Weight ≤ 2000 g (47)			Fetal weight > 2000 g (150)			
	Nulliparity (33)	Multiparity (14)		Nulliparity (81)	Multiparity (69)		
PSemw	4.5 ± 2.4 mm	4.3 ± 2.7 mm	H = 0.32 *p* = 0.57	5.0 ± 3.1 mm	4.7 ± 2.8 mm	H = 0.64 *p* = 0.42	*p* > 0.05
PSw	4.7 ± 2.6 mm	6.3 ± 2.9 mm	H = 0.01 *p* = 0.97	5.8 ± 2.9 mm	6.4 ± 3.6 mm	H = 0.04 *p* = 0.83	*p* > 0.05
PSd	13.4 ± 4.7 mm	15.0 ± 4.7 mm	H = 0.57 *p* = 0.45	13.3 ± 4.9 mm	14.6 ± 4.8 mm	H = 1.56 *p* = 0.21	*p* > 0.05
PT-PT	**15.9 ± 7.2 mm**	**13.4 ± 6.2 mm**	**H = 8.66** *p* = 0.03 *	14.3 ± 5.9 mm	16.6 ± 7.6 mm	H = 0.98 *p* = 0.32	*p* = 0.03 *

* Kruskal–Wallis one-way analysis of variance by ranks/H/; statistically significant *p*-values are shown in bold.

## Data Availability

The data that support the findings of this study are available from the corresponding author upon reasonable request.

## Supplementary Materials

The following supporting information can be downloaded at: https://www.mdpi.com/article/10.3390/jcm14113898/s1, Short video presentation: PSw; PT-PT measurements.

## Abbreviations

The following abbreviations are used in this manuscript:

PS	Pubic symphysis
US	Ultrasonography
PSemw	Pubic symphysis entry middle width
PT-PT	Intertubercular distance
PSw	Pubic symphysis width
PSd	Pubic symphysis depth
